# Supplementary material for: Exploring the phenomenon of intrusive mental imagery after suicide bereavement: A qualitative interview study in a British sample
Source: PLoS One. 2023 Aug 17;18(8):e0284897. doi: 10.1371/journal.pone.0284897 (PMC10434947; doi:10.1371/journal.pone.0284897)
Supplement: S1 Appendix — (DOCX) [file pone.0284897.s001.docx]

**S1 Appendix: Topic guide**

Semi-structured interview topics/key questions:

Opening statement, e.g., “You told us in the questionnaire that you lost your X due to suicide X years ago, and that he/she was X years old at the time” …

- How did you find out about the death? Were there aspects of it that you did not find out about until much later?
- Were you aware of any circumstances around the death? For example, financial reasons, mental health, significant life event(s)
- Did you have any warning signs that he/she was considering suicide? E.g., expressing suicidal thoughts, previous attempts, comments on social media, becoming very withdrawn.
- How frequently do you experience images relating to the suicide?
- How much control do you feel that you have over the experience of these images?
- Can you tell me a little more about the content of the images you experience?
- You mentioned that s/he had died using (insert method), to what extent does this feature in the images you experience?
- When you experience these image(s), how do you feel?
- When you experience these image(s), what does this mean to you?
- When you experience these image(s), how do you react or behave?
- When you experience these image(s), how do you cope?
- Do you find these images helpful or comforting in any way?
- Do you find these images unhelpful or distressing in any way?
- Are these images related to the deceased only or do you feature in the images in some way?
- Is there any other way in which these images impact upon you positively or negatively?
- If you no longer had these images, would you miss them or feel relieved?
