# Supplementary material for: Exploring the phenomenon of intrusive mental imagery after suicide bereavement: A qualitative interview study in a British sample
Source: PLoS One. 2023 Aug 17;18(8):e0284897. doi: 10.1371/journal.pone.0284897 (PMC10434947; doi:10.1371/journal.pone.0284897)
Supplement: S2 Appendix — (DOCX) [file pone.0284897.s002.docx]

**S2 Appendix: Distribution of themes across 18 interview transcripts**

|  | P1 | P2 | P3 | P4 | P5 | P6 | P7 | P8 | P9 | P10 | P11 | P12 | P13 | P14 | P15 | P16 | P17 | P18 | (*N*) |
| --- | --- | --- | --- | --- | --- | --- | --- | --- | --- | --- | --- | --- | --- | --- | --- | --- | --- | --- | --- |
| **1. Characteristics of intrusive imagery** |  |  |  |  |  |  |  |  |  |  |  |  |  |  |  |  |  |  |  |
| **1.1. Nature of imagery** | ▲ | ▲ | ▲ | ▲ | ▲ | ▲ | ▲ | ▲ | ▲ | ▲ | ▲ | ▲ | ▲ | ▲ | ▲ | ▲ | ▲ | ▲ | 18 |
| 1.1.1 Discovering the deceased |  |  | ◆ | ◆ |  |  |  |  |  | ◆ | ◆ |  |  |  |  |  | ◆ |  | 5 |
| 1.1.2 Suicide method as a key feature | ◆ | ◆ | ◆ |  | ◆ | ◆ | ◆ | ◆ | ◆ |  | ◆ | ◆ | ◆ | ◆ | ◆ | ◆ | ◆ |  | 15 |
| 1.1.3 Only the deceased is present in imagery | ◆ | ◆ |  |  |  | ◆ | ◆ |  | ◆ | ◆ | ◆ | ◆ |  |  |  | ◆ |  |  | 9 |
| 1.1.4 Picturing the deceased as dead | ◆ | ◆ | ◆ | ◆ | ◆ |  | ◆ | ◆ | ◆ | ◆ | ◆ |  | ◆ |  | ◆ | ◆ | ◆ | ◆ | 15 |
| 1.1.5 Piecing together what happened at the scene of the suicide | ◆ |  |  | ◆ |  | ◆ | ◆ |  | ◆ |  | ◆ | ◆ | ◆ | ◆ | ◆ | ◆ | ◆ |  | 12 |
| 1.1.6 Questioning |  | ◆ |  | ◆ |  | ◆ | ◆ |  |  |  |  |  | ◆ | ◆ | ◆ |  | ◆ |  | 8 |
| **1.2. Degree of control over imagery** | ▲ | ▲ | ▲ | ▲ | ▲ | ▲ | ▲ | ▲ | ▲ | ▲ | ▲ | ▲ | ▲ | ▲ | ▲ | ▲ | ▲ | ▲ | 18 |
| 1.2.1 Lack of control | ◆ | ◆ | ◆ | ◆ | ◆ | ◆ |  | ◆ | ◆ | ◆ | ◆ | ◆ | ◆ | ◆ | ◆ | ◆ | ◆ | ◆ | 17 |
| 1.2.2 Triggers for imagery | ◆ | ◆ | ◆ |  |  |  | ◆ | ◆ |  | ◆ | ◆ | ◆ | ◆ | ◆ |  |  | ◆ |  | 11 |
| **1.3. Changes in imagery over time** | ▲ | ▲ | ▲ | ▲ | ▲ | ▲ |  |  | ▲ |  | ▲ | ▲ |  |  | ▲ | ▲ |  |  | 11 |
| 1.3.1 Reduced impact | ◆ | ◆ | ◆ | ◆ | ◆ | ◆ |  |  | ◆ |  | ◆ | ◆ |  |  | ◆ | ◆ |  |  | 11 |
| 1.3.2 The influence of control | ◆ |  |  |  |  | ◆ |  |  | ◆ |  | ◆ |  |  |  |  |  |  |  | 4 |
| **2. Emotional, cognitive and behavioural responses to intrusive imagery** |  |  |  |  |  |  |  |  |  |  |  |  |  |  |  |  |  |  |  |
| **2.1. Imagery as unhelpful and distressing** | ▲ | ▲ |  | ▲ |  | ▲ | ▲ | ▲ | ▲ | ▲ | ▲ | ▲ | ▲ |  | ▲ | ▲ | ▲ |  | 14 |
| 2.1.1 Imagery as distressing | ◆ | ◆ |  | ◆ |  | ◆ | ◆ | ◆ | ◆ | ◆ | ◆ | ◆ |  |  | ◆ | ◆ | ◆ |  | 13 |
| 2.1.2 Intrusive imagery interferes with positive memories and images of the deceased |  |  |  | ◆ |  |  | ◆ | ◆ |  |  | ◆ |  | ◆ |  | ◆ | ◆ |  |  | 7 |
| 2.1.3 Fabricated imagery as unhelpful and impeding closure | ◆ |  |  |  |  | ◆ |  | ◆ | ◆ |  |  |  |  |  |  |  | ◆ |  | 5 |
| **2.2. Impact of imagery** | ▲ | ▲ | ▲ | ▲ | ▲ | ▲ | ▲ | ▲ | ▲ | ▲ | ▲ | ▲ | ▲ | ▲ | ▲ | ▲ | ▲ |  | 17 |
| 2.2.1 The negative impact of imagery | ◆ | ◆ |  | ◆ | ◆ |  |  | ◆ | ◆ | ◆ |  | ◆ | ◆ |  | ◆ | ◆ |  |  | 11 |
| 2.2.2 Physical impact | ◆ | ◆ | ◆ | ◆ | ◆ | ◆ |  |  | ◆ |  |  | ◆ |  |  |  | ◆ |  |  | 9 |
| 2.2.3 Mixed emotional responses to imagery | ◆ |  |  | ◆ | ◆ |  | ◆ | ◆ | ◆ | ◆ | ◆ |  | ◆ | ◆ | ◆ |  | ◆ |  | 12 |
| **2.3. Ways of coping** | ▲ | ▲ | ▲ |  |  | ▲ |  | ▲ | ▲ |  | ▲ |  |  |  | ▲ | ▲ | ▲ |  | 10 |
| 2.3.1 Distraction |  |  | ◆ |  |  |  |  | ◆ | ◆ |  | ◆ |  |  |  | ◆ |  |  |  | 5 |
| 2.3.2 Overwriting imagery | ◆ | ◆ |  |  |  | ◆ |  | ◆ | ◆ |  |  |  |  |  |  | ◆ |  |  | 6 |
| 2.3.3 Attempts to avoid imagery |  | ◆ | ◆ |  |  | ◆ |  | ◆ | ◆ |  |  |  |  |  | ◆ | ◆ | ◆ |  | 8 |
| **2.4. Positive aspects of the experience of imagery** | ▲ | ▲ | ▲ | ▲ | ▲ | ▲ | ▲ | ▲ | ▲ | ▲ | ▲ | ▲ | ▲ | ▲ | ▲ |  | ▲ | ▲ | 17 |
| 2.4.1 Post-traumatic growth | ◆ |  | ◆ | ◆ |  |  |  |  |  |  | ◆ | ◆ |  |  |  |  | ◆ |  | 6 |
| 2.4.2 Imagery as supporting processing | ◆ | ◆ |  |  | ◆ | ◆ | ◆ | ◆ | ◆ |  | ◆ |  | ◆ | ◆ |  |  | ◆ | ◆ | 12 |
| 2.4.3 Imagery as important regardless of the distress |  |  | ◆ |  |  |  |  |  |  | ◆ |  | ◆ |  |  |  |  | ◆ |  | 4 |
| 2.4.4 Imagery as facilitating a connection with the deceased |  |  | ◆ | ◆ |  |  |  |  |  |  | ◆ |  | ◆ | ◆ | ◆ |  |  | ◆ | 7 |
| 2.4.5 Fear of erasing the memory of the deceased |  |  |  |  |  | ◆ |  |  |  | ◆ |  | ◆ | ◆ | ◆ |  |  | ◆ |  | 6 |
